# The Development of a New Mental Health Pre‐Registration Nursing Curriculum: Reclaiming Professional Identity and Field Specific Content

**DOI:** 10.1111/inm.70255

**Published:** 2026-04-07

**Authors:** Jane Fisher, Simon Baverstock, Gareth Bell, Jayne Firestone, April Ingleby, Julie McCartney, Eve Potts, Diane Roberts, Michael Smith, Donna Steele

**Affiliations:** ^1^ University of Lancashire Preston UK

**Keywords:** critical thinking, genericism, heutagogy, mental health nurse education, NMC standards

## Abstract

This paper outlines the design and development of a 3‐year undergraduate pre‐registration mental health nursing curriculum in the United Kingdom. It resists the trend towards generic nurse education, reclaims mental health field‐specific identity and equips future nurses with the knowledge, skills and values to meet the complex mental health needs of contemporary populations. International literature reflects growing concern over the dilution of mental health nursing knowledge and the erosion of professional identity, largely attributed to generic curriculum models and standardised regulatory frameworks. Critics argue that the dominance of adult‐centric education compromises field‐specific learning, perpetuating a theory–practice gap and leaving graduates underprepared for mental health practice. Calls for reform have intensified globally, yet practical examples of curriculum redesign to address these issues remain limited. Issues were approached through a comprehensive review and redesign of the current mental health nursing curriculum considering relevant pedagogical theory and international critiques of genericism in nursing education. The authors, as academic leads for the curriculum redesign, embedded field‐specific modules, innovative assessments, restorative supervision and mental health‐focused simulation. Heutagogy informed the pedagogical framework, aiming to produce self‐determined, critically reflexive practitioners. The redesigned curriculum challenges genericism and reinstates mental health‐specific content, with assessments and simulations contextualised to authentic practice. This approach strengthens professional identity, reduces reliance on artificial intelligence generated work through dialogical assessments and promotes critical thinking essential for ethical, person‐centred care. Future evaluation will focus on student retention, satisfaction and preparedness for practice.

## 1 Aims

The aim of this perspective paper is to outline the design and development of a new pre‐registration mental health nursing curriculum. The paper will explore how the curriculum design resists the trend towards generic nurse education, reclaims mental health field‐specific identity and equips future nurses with the knowledge, skills and values to meet the complex mental health needs of contemporary populations.

## 2 Background

Mental illness is a global health concern, and the worldwide leading cause of disability, yet, on average, countries dedicate < 2% of their healthcare budgets to mental health care provision (World Health Organization 2022). This renders services chronically under‐funded and under‐resourced. Mental health nursing represents the largest professional group involved in delivering care. Despite this vital role, international nurse education has been called out as not fit for purpose, with leading academics contending standards of mental health care may fall unless urgent action is taken (Bifarin et al. 2024; Hurley and Ramsay 2008; Warrender et al. 2023). Posited against a backdrop of growing global dissatisfaction with mental health nurse education, we encourage other Higher Education Institutions (HEIs) to rebel against a generic nurse curriculum, and to reclaim our professional identity.

The authors of this perspective paper are situated in The School of Nursing and Midwifery at the University of Lancashire, and the school offers all four fields of nursing (adult, children and young people, mental health and learning disability nursing). As part of a full pre‐registration nursing curriculum redesign, the authors were supported by senior leadership at the University of Lancashire to rewrite all 3 years of the undergraduate mental health nursing curriculum. In September 2024, years one and two of the new curriculum were introduced, whilst existing third years stayed on the old curriculum. Year three of the new curriculum commenced in September 2025. Mental health nursing has two intakes of direct entry students per year and one intake per year of Registered Nurse Degree Apprenticeship (RNDA), with approximately 300 students across all 3 years. We deliver 80% of module teaching face to face, with 20% scheduled asynchronous learning.

## 3 Design

This paper is presented as a position paper offering a reflexive exploration of the design and implementation of a new mental health nursing curriculum and its underpinning theoretical and pedagogical philosophy.

## 4 Method

This perspective paper was developed through ongoing reflexive discussions on the design and implementation of a new curriculum undertaken by the authors and the wider academic teaching team. Issues were approached through a comprehensive review and redesign of the current mental health nursing curriculum considering relevant pedagogical theory and international critiques of genericism in nurse education. The authors, as academic leads for the curriculum redesign, embedded field‐specific modules, innovative assessments, restorative supervision and mental health‐focused simulation. Heutagogy informed the pedagogical framework, aiming to produce self‐determined, critically reflexive practitioners.

## 5 Curriculum Reforms and the Rise of Genericism

The report into the Mid Staffordshire Hospital (Francis 2013) drastically disturbed the state of nursing and healthcare in the United Kingdom (UK), with erroneous failings in patient care triggering both public and professional outcry. Health Education England (HEE) and the Nursing and Midwifery Council (NMC) subsequently commissioned the Willis Report (2015) to shape the future of nursing and healthcare, aiming to prevent similar future crises. Key recommendations focused on nursing flexibility, adaptability and shared competencies across all four nursing fields (adult, mental health, learning disability, and children and young people's nursing). The report interrogated the current model of nurse education, questioning its suitability for the physical health demands of the population (Willis 2015). A nursing curriculum based on 2 years of shared generic modules and 1 year of field‐specific content was recommended and extensively adopted, despite being non‐mandatory.

Following the Willis Report, the NMC published their updated standards for nurse education with one set of proficiencies for all four fields of nursing (Nursing and Midwifery Council 2018). This marked a paradigm shift in UK nursing where adult physical health‐based competencies were favoured over mental health‐specific skills (Connell et al. 2022).

## 6 Impact on Mental Health Nursing Identity

While intended to improve adaptability and meet the physical health care needs of the population, the updated NMC standards for education have drawn fierce criticism (Warrender et al. 2023). Mental health nursing competencies were diluted (Connell et al. 2022) and the lack of field‐specific guidance reinforced mental health nursing as a subordinate discipline (Bifarin et al. 2024). This threatens not only our professional identity but also the quality of mental health care delivery (Fisher 2025). Similar concerns have been raised by other smaller nursing fields who argue that adult nursing perspectives have dominated curriculum development, thus marginalising smaller fields (Glasper and Fallon 2021).

## 7 Global Critique of Generic Nurse Education

Concerns over the erosion of mental health nursing identity within pre‐registration education are not isolated to the United Kingdom. Global evidence highlights an increasing shift towards generic nurse education models, raising fears of a loss of specialist knowledge and skills and a negative impact on patient care. Australia operates on a generic undergraduate nursing education, with impassioned calls for the reintroduction of specialised programmes (Lakeman et al. 2023; Stephenson 2017). International patient voices also advocate for field‐specific training, warning that genericism undermines safe, person‐centred mental health care (Hurley et al. 2023).

## 8 Grassroots Advocacy and Calls for Change

Mounting dissatisfaction has given rise to a grassroots movement (Mental Health Deserves Better 2022), whose manifesto challenges the perceived erosion of mental health nursing identity. In an open letter to the NMC, the group warned of the consequences of diluting specialist skills and demanded a review of educational standards (Mental Health Deserves Better 2023). While the NMC reaffirmed its commitment to all four fields, it delegated responsibility for curriculum interpretation to HEIs (Nursing and Midwifery Council 2023). This creates significant variation and raises questions about the consistency of mental health nurse education and preparation for practice (Warrender et al. 2023).

Against this backdrop of regulatory ambiguity and professional concern, this paper responds with a practical solution: a curriculum redesign aimed at reclaiming mental health nursing identity and embedding field‐specific knowledge throughout the student journey.

## 9 Pedagogical Approach

The overarching pedagogical philosophy of the new curriculum is the production of lifelong critical thinking nurses, possessing the skills and ability to adapt to an ever‐evolving healthcare landscape. Heutagogy is an extension to andragogy; a pedagogical approach to education focusing on self‐determined learners (Hase and Kenyon 2000). This philosophy cements the onus and motivation on students to take charge of their own learning. There is an evolving evidence base for heutagogy as an exemplar approach to contemporary health care education, and its creation of digital self‐motivated learners equipped for the modern workplace (Gillaspy and Vasilica 2021). As an antithesis to the traditional ‘chalk and talk’ method, where educators control the flow of knowledge, heutagogy takes a different approach, allowing the learner choice and independence.

This is not without challenge, potentially creating an uneasy dynamic within nurse education, which is heavily dictated by regulated competencies and prescribed learning from governing bodies. Student expectations of their education present additional barriers, with some students assimilating to the archetype passive learner, expecting to be spoon‐fed the required knowledge. This radical departure from passive‐based learning can be challenging to students; therefore, the new curriculum focuses on supporting learners to evolve from passive to active learners in year one. Year two focuses on the transition from active to self‐determined learners. Finally, in year three, the ideology of a self‐determined learner is embedded. Blaschke and Hase (2016) emphasise the importance of the transition from a passive learner to an analyst and synthesiser, vital skills for the twenty‐first century learner and healthcare professional.

## 10 Assessment Strategies

Authenticity is a widespread fundamental pedagogical tenet; however, there are multifaceted challenges in translating this to the assessment of nursing students (Maude et al. 2021). Absent in the author's previous curriculum incarnation, the redesign allowed for the creation of innovative real‐word assessment strategies. In line with our previously discussed pedagogical approach, these assessment are based on principles of heutagogy (Gillaspy and Vasilica 2021), whilst attempting to bridge the gap between nursing theory and practice (Warrender 2022a). The theory–practice gap is a longstanding concern within nurse education, manifested when academic learning is viewed as superfluous to the more practical learning received on clinical placements. Warrender (2022a) references the evocative concept of nurses ‘eating their young’. This phenomenon describes forcing new nurses (and arguably student nurses) to assimilate into practice conditions, abandoning their theoretical nursing knowledge. This threatens to fragment and jeopardise not only the integration of students' learning, but the evolution of contemporary evidence‐based practice.

Assessment strategies in the author's previous curriculum focused on anonymous written submissions. These were viewed by students as detached from their practical‐based learning. Failing to see the value of theoretical assessment affected both students' academic attainment and engagement with theory. Assessment feedback was anonymised and untimely, resulting in poor engagement with the developmental advice. An additional global contributing factor is artificial intelligence (AI), which is infiltrating both academia and the workplace at a remarkable rate. Detection software has been developed to support HEIs in the detection of artificially generated academic work. However, its accuracy is debated, plus it has the potential for bias in minority groups (Alexander et al. 2023; Jiang et al. 2024). Therefore, to embrace principles of heutagogy, real‐world assessments, and to avoid AI, a paradigm shift in assessment strategy was required. The existing outdated assessments were remodelled into real‐world, dynamic methods of assessment fit for future mental health nurses.

### 10.1 Person‐Centred Care Plan

A new assessment requires students to create a person‐centred, evidence‐based care plan for managing pharmacological interventions for a mental health condition, alongside any associated co‐morbid health conditions. This must consider the patient's wishes and relevant legal frameworks. A 15‐min professional dialogue with academic staff follows, aimed at carefully assessing the content and justification of the care plan. With a clear connection to clinical practice, this assessment addresses the poor physical health of patients prescribed psychiatric medications (NHS England 2024), thus narrowing the theory–practice divide. Crucially, stakeholder feedback from patients and carers was affirmative for this assessment method, and the collaborative person‐centred approach to care planning was valued. The professional dialogue aspect of the assessment mitigates the risk of students employing AI in their written work, thus allowing educators to fully assess the student's comprehension and emerging competence.

### 10.2 Person Centred Formulation

A further pioneering assessment approach involves the creation of a person‐centred formulation, incorporating relevant psychological theories. Students must show evidence of co‐production with a patient and the preservation of the patient's voice throughout. This will lead to the recommendation of a non‐pharmacological nurse‐led intervention, again maintaining evidence of co‐production. This module also incorporates group work as part of the assessment strategy, which mimics multidisciplinary team working. Students will question any assumptions made in the formulation and engage in a critical discussion about the suitability of the chosen nurse‐led intervention. Collaborative or group assessments can evoke troubling emotions in students (Allan 2016; Traill 2023), yet there are evidence‐based benefits, particularly for nursing students who require effective team working skills for the workplace. Again, these contemporary assessment strategies aim to bridge students' perception of the dreaded theory–practice gap.

### 10.3 Professional Reflexive Discussions

Practice modules are now assessed via a professional discussion about the students' progress and learning on clinical placement. Previously, this was assessed through a reflexive written assignment constricted by models and frameworks. The reflexive discussion allows academic staff a more realistic and flexible structure to determine students' genuine learning and development, whilst removing the students' desire to pass a written anonymised assignment. Discussions will be highly personalised and meaningful, whilst still requiring students to engage with evidence and literature to explore their knowledge base and reflexive learning journey.

A disadvantage of moving towards the above collaborative and face‐to‐face assessments is the removal of anonymity and the potential for bias. However, it does offer the opportunity to provide meaningful and personalised feedback. Students' previous experience of education, socioeconomic background, family history of higher education and individual traits all impact students' journey to academic success. Educational baggage stemming from past learning experiences is vital to acknowledge in supporting students to engage in meaningful feedback (Traill 2023).

The contemporary emphasis on meaningful and timely (live and face to face) formative feedback is reflected in the pedagogical philosophy of the new curriculum. Modules now prioritise formative feedback rather than traditional group assignment support, based on a question‐and‐answer format. By default, this prioritised passing the assignment as opposed to fostering self‐development and self‐motivation in alignment with principles of heutagogy (Blaschke and Hase 2016).

### 10.4 Written Assignments

Despite the ongoing risk of students engaging with AI to complete their academic work, it remains imperative that future nurses can produce comprehensive written documents. Therefore, in year three, the theory module defaults back to a traditional written submitted assignment. This focuses on the student's ability to justify a complex and ethical clinical decision that they will likely encounter in their role as a newly registered mental health nurse. Mental health nurses navigate challenging ethical and moral dilemmas and values conflict (Connell et al. 2022). The ability to engage in morally and ethically critical thinking is imperative for future mental health nurses (Adam and Juergensen 2019). They need to consider the nuances and complexities of clinical decisions and articulate a strong evidence‐based justification for the actions of the multidisciplinary team. Gone are the days when nurses are the handmaidens of biomedical psychiatry. We are educated professionals (Judge and Fisher 2024), with the ability to challenge coercive and punitive practices, whilst amplifying the voice of the patient and their loved ones. This requires advanced critical thinking.

### 10.5 Practice Modules

Mental health nurse education in the United Kingdom has an equal split between theory and practice hours. The theory–practice gap has already been highlighted as problematic (Warrender 2022a). In the former curriculum, students departed university for extended periods to complete their clinical placements and meet the standardised practice requirements. These have been posited as prioritising adult‐based nursing procedural competencies, thus compounding the philosophical shift towards a generic nurse (Warrender et al. 2023).

The paradigm shifts in the redesigned practice modules facilitated a rebellion against chasing clinical proficiencies, many of which are viewed as irrelevant to mental health nursing care (Warrender 2022b). However, a pragmatic approach is required, as practice assessment requirements dictated by regulatory bodies remain a required element in the competencies of a registered nurse. As such, this element remains a mandatory component of assessment in the practice modules.

## 11 Keeping in Touch (KIT) Days

The curriculum redesign embedded structured group reflexivity within the practice period. These take the form of Keeping in Touch (KIT) days, occurring weekly in university, for the duration of clinical placements. Teaching will be with the student's personal tutor group and a regular facilitator. Each 6‐h KIT day will explore fundamental elements essential to the role of a contemporary mental health nurse. These include professionalism, therapeutic relationships, advanced interpersonal and communication skills, team working, advocacy and the ability to support people in acute distress. By simultaneously providing structured reflexivity alongside clinical placements, it creates authentic and meaningful learning experiences. Students can collaboratively reflect within a supportive, safe and regulated environment. There is no intention that these sessions will replace the invaluable work of practice assessors, supervisors and the wider practice development teams in clinical placements. They add a thoughtful layer of restorative supervision and reflexive learning.

KIT days have been carefully structured to ensure students are not consumed by negative experiences. The skill and clinical insight of the facilitator, being an experienced mental health nurse academic, ensures that students are supported to process challenges and construct solutions without becoming overwhelmed or consumed by negative experiences. The underpinning theory behind the KIT days is restorative supervision. This is a structured, supportive approach that prompts reflection on the emotional impact of the role of a nurse (Wallbank 2016). It aims to restore well‐being, build resilience and prevent burnout (NHS England 2021). Using a safe, non‐judgemental space, students can explore challenges, process difficult experiences and strengthen their professional identity. With the added opportunity for peer support, a variety of strategies for navigating challenging situations common to a mental health practice environment can be explored. This fosters an environment where students can develop self‐awareness and discover strategies for resilience and mental well‐being. Whilst attending clinical placements, student nurses must balance full‐time placement hours whilst also preparing for assignments, managing work–life balance, and potential personal caring responsibilities alongside other protected characteristics. KIT days are designed as a weekly haven, allowing students the opportunity to consider the real‐life experiences and challenges of meeting the mental health needs of the population, alongside the maintenance of their own well‐being.

This innovative approach to practice modules within mental health nurse education aligns with principles of heutagogy and the development of independent, initiative‐taking learners with a powerful sense of mental health nursing identity. This will foster enthusiastic future mental health nurses with longevity and, although a much‐disputed concept, resilience (Fisher and Jones 2023, 2025).

## 12 Patient Involvement

Patients and carers have been involved at each step of the wider nursing curriculum development. However, the concept of co‐production faces fierce criticism from patient survivor literature. For example, there is a high risk that patient stories are co‐opted by those with social and academic dominance (Russo 2023), and the patient voice overwritten by dominant narratives within academic institutions (Russo and Beresford 2015). The lead author of this paper has lived through tokenistic co‐production activities and its damaging aftereffects (Fisher 2024). Merely inviting someone into the classroom to share their story and leave devalues other knowledge and skills. Therefore, within the mental health practice modules, the university patient and carer group have been granted autonomy to design their own sessions. This allows patients and carers a direct voice to express their collective views on what qualities and values mental health nurses need to meaningfully and professionally advocate and care for their patients. This aims to achieve more genuine co‐production and co‐writing curriculum content.

## 13 Mental Health‐Specific Theory Modules

During the redesign of the nursing curriculum, the authors fiercely advocated for mental health‐specific theory modules present across all 3 years of nurse education. Module titles are engaging, whilst instilling a sense of belonging. Entitled ‘So you want to be a mental health nurse?’ the year one theory module embeds mental health nurse identity, theory and unique skill‐set from the onset of students' education. This equips students for their first mental health clinical placement. The previous curriculum model resulted in students attending mental health clinical placements and working alongside vulnerable people with complex mental health needs, with no foundational mental health‐specific education or knowledge base. By designing a curriculum that adequately equips students for clinical practice will improve students' confidence and competence and will be eagerly evaluated.

### 13.1 Critical Thinking

The year three theory module aims to embed prior learning whilst honing students' critical thinking and reflexivity skills. With the intention of equipping students for clinical practice and their first mental health nursing post, module learning outcomes focus on making and justifying complex ethical clinical decisions. Criticality is a vital skill for mental health nurses (Cleary et al. 2023) who often operate within complex legal, moral and ethical parameters. With a legacy of paternalism and coercion, fierce criticisms aimed at psychiatry, a contended evidence base for interventions, and diagnosis itself, mental health nurses require advanced critical thinking and decision‐making ability. Students will be exposed to both the psychiatric horrors of the past and present, and challenged to examine their professional and personal values. Topics such as systemic racism within mental health services, the role of the nurse in iatrogenic harm and epistemic injustice (Fisher 2023) will be faced head‐on, all designed to produce nurses with the confidence and skills to forge a new way forward in mental health care.

## 14 Simulation Modules

The ideology of simulated practice within nurse education is to replicate real world clinical environments, using a range of technology, resources and scenarios (Nursing and Midwifery Council 2024). This safe and patient‐free environment allows students to perform clinical skills and competencies away from direct patient care. Students are confronted with new experiences, triggering reflexivity and critical analysis in relation to their role as a student mental health nurse. The identification of knowledge deficits is encouraged. In alignment with Kolbs' experiential learning theory (Kolb 1984), the Nursing and Midwifery Council (2024) emphasise there should be opportunity for repetition, feedback, and evaluation of the development of clinical skills in a simulated environment. Within this carefully constructed evaluation, students formulate meaning and personal goals within the framework of experiential learning.

Simulation has developed at an exponential rate over the last decade. Technology is extremely sophisticated with the continuous development of high‐fidelity manikins mimicking the full range of physiological and pathophysiological functions. Additional simulated technology now incorporates virtual reality (VR) headsets and immersive reality (IR) specialised suits. These sophisticated technologies provide an immersive environment, heightening students' senses and allowing them to consider the patients' perspective (Slater et al. 2021). This alongside specialised garments and suits which impact on mobility and senses, creates an authentic patient experience aimed to generate empathy and compassion (Mandegari Bamakan et al. 2021).

During the global chaos of COVID‐19, nursing students were removed from direct clinical practice. The impact of the unprecedented pandemic altered international nurse education, and the use of simulated learning became a necessity to train new nurses. The pressure to navigate the pandemic, whilst achieving national targets for newly qualified nurses resulted in a paradigm shift in nurse education. In the United Kingdom, this resulted in the introduction of simulated placements, where HEI's can deliver up to 600 h of simulated learning across a 3‐year programme (Nursing and Midwifery Council 2024). This remains in place following COVID‐19, permitting HEI's creativity in the development of simulated learning.

The curriculum redesign has permitted the development of innovative mental health field‐specific simulated learning packages, which were largely absent in the previous curriculum. These are now embedded from year one, where students attend a field‐specific simulated placement. They follow the care of a range of patients admitted to an acute inpatient unit. Mandated tasks include mental state examination, risk assessments and physical healthcare monitoring. Facilitators encourage teamwork and professionalism within the multidisciplinary team, whilst embedding underpinning values of compassionate person‐centred care and shared decision‐making. Throughout this simulated placement, students are prompted to explore challenging issues around capacity, consent to treatment, safeguarding and ethical issues. This rich, varied and most importantly mental health‐specific simulated placement provides a solid foundation on which students can develop mental health‐specific nursing skills. The inclusion of gamified learning results in a memorable and impactful simulated learning experience.

Mental health‐specific simulation continues into the second and third years of the redesigned curriculum. Developed in‐house in collaboration with frontline clinicians, our immersive reality scenario offers a powerful learning experience focused on electroconvulsive therapy (ECT). The simulation was filmed in the local trust's ECT suite and includes all relevant healthcare professionals. Because it would be ethically inappropriate to film an actual patient receiving ECT, a nurse takes the role of the patient, without undergoing treatment. After viewing the scenario, students prepare a patient for ECT and complete a clinical handover to the ECT team. They then provide post‐ECT and post‐anaesthesia care. Within the skills labs, students work with three simulated patients to consolidate these competencies. As a controversial mental health treatment generating heated classroom discussions, this simulated learning package provides a clinical context to all students. This was achieved by filming within the local trust's ECT department. Additionally, building on the skills developed in year one, students will return to the inpatient mental health ward, where they will be introduced to mental state assessment and risk management skills. They will be required to demonstrate advanced interpersonal skills and the therapeutic use of self, whilst managing risks such as self‐harm, patient agitation and acute distress. Students will be exposed to a coroner's court scenario, where they will be required to write a report, give evidence and demonstrate critical decision‐making in alignment with legal and ethical frameworks.

As previously discussed in this paper, the NMC outline procedural skills that all UK nurses, in all fields, must demonstrate. These include nasogastric tubes, blood transfusions, venepuncture and cannulation. Such skills are now taught within a mental health context, addressing the theory–practice gap and contextualising clinical skills within a mental health nursing clinical setting. For example, venepuncture and cannulation, catheter care and the management of IV fluids are taught as part of the ECT immersive suit. Nasogastric tubes will be explored within the context of eating disorders, giving a clear mental health clinical context to prescribed competencies.

## 15 Conclusion

This perspective paper has outlined a curriculum redesign which has resisted the international genericism of mental health nurse education, whilst reclaiming mental health field‐specific identity. New assessment strategies have allowed for personalised and timely feedback embracing the crucial relational elements of effective student feedback. Innovative and creative assessments align to principles of heutagogy, whilst avoiding unauthorised use of AI. The incorporation of restorative supervision and KIT days within practice modules encourages a move from chasing competencies to meaningful and personalised learning and development. Mental health theory modules in each year regularly expose students to field‐specific content, combining to produce reflexive critical thinking nurses. Simulation technology has been embraced, including the use of gamification, virtual and immersive reality, all designed around mental health‐specific scenarios.

Future research is needed to evaluate the impact of the new curriculum on retention, attainment, student satisfaction and preparedness for mental health nursing practice. We implore other HEI's to reclaim our professional identity and redress the balance of field‐specific content.

## 16 Relevance for Clinical Practice

Producing nurses who are confident, critically reflective and equipped with mental health‐specific competencies is essential for safe, effective and person‐centred nursing care. By embedding identity formation, ethical decision‐making and authentic field‐specific clinical skills from year one of undergraduate study, this curriculum better prepares nurses to address patient needs, challenge coercive practices and deliver high‐quality care in a complex mental health landscape.

## Funding

The authors have nothing to report.

## Conflicts of Interest

The authors declare no conflicts of interest.

## Data Availability

Data sharing is not applicable to this article as no datasets were generated or analysed during this study.
